# Identification and Quantification of Polycyclic Aromatic Hydrocarbons in Polyhydroxyalkanoates Produced from Mixed Microbial Cultures and Municipal Organic Wastes at Pilot Scale

**DOI:** 10.3390/molecules26030539

**Published:** 2021-01-21

**Authors:** Chiara Cavaliere, Anna Laura Capriotti, Andrea Cerrato, Laura Lorini, Carmela Maria Montone, Francesco Valentino, Aldo Laganà, Mauro Majone

**Affiliations:** 1 Department of Chemistry, Università di Roma “La Sapienza”, Piazzale Aldo Moro 5, 00185 Rome, Italy; chiara.cavaliere@uniroma1.it (C.C.); andrea.cerrato@uniroma1.it (A.C.); laura.lorini@uniroma1.it (L.L.); carmelamaria.montone@uniroma1.it (C.M.M.); francesco.valentino@uniroma1.it (F.V.); aldo.lagana@uniroma1.it (A.L.); mauro.majone@uniroma1.it (M.M.); 2 CNR NANOTEC, Campus Ecotekne, University del Salento, Via Monteroni, 73100 Lecce, Italy

**Keywords:** biopolymers, municipal waste, sewage sludge, contaminants, pollutants

## Abstract

Polyhydroxyalkanoates (PHAs) are well-known biodegradable plastics produced by various bacterial strains, whose major drawback is constituted by the high cost of their synthesis. Producing PHAs from mixed microbial cultures and employing organic wastes as a carbon source allows us to both reduce cost and valorize available renewable resources, such as food waste and sewage sludge. However, different types of pollutants, originally contained in organic matrices, could persist into the final product, thus compromising their safety. In this work, the exploitation of municipal wastes for PHA production is evaluated from the environmental and health safety aspect by determining the presence of polycyclic aromatic hydrocarbons (PAHs) in both commercial and waste-based PHA samples. Quantification of PAHs by gas chromatography-mass spectrometry on 24 PHA samples obtained in different conditions showed very low contamination levels, in the range of ppb to a few ppm. Moreover, the contaminant content seems to be dependent on the type of PHA stabilization and extraction, but independent from the type of feedstock. Commercial PHA derived from crops, selected for comparison, showed PAH content comparable to that detected in PHAs derived from organic fraction of municipal solid waste. Although there is no specific regulation on PAH maximum levels in PHAs, detected concentrations were consistently lower than threshold limit values set by regulation and guidelines for similar materials and/or applications. This suggests that the use of organic waste as substrate for PHA production is safe for both the human health and the environment.

## 1. Introduction

Among the various types of biodegradable plastics, polyhydroxyalkanoates (PHAs) are the most known, being recognized as completely biocompatible and biodegradable. They are microbial polyesters produced by a wide range of microorganisms, mostly as intracellular storage compounds for energy and carbon [1]. Their properties span a wide range, including thermoplastic, mechanical and electrometric properties. However, industrial-scale production is still based on pure cultures that require sterile conditions and synthetic substrates. Currently, the interest is focused on combining mixed microbial cultures (MMC) and organic feedstocks as solid waste and wastewater for PHA pilot-scale production [2,3]. Indeed, renewable materials from microorganisms can provide a source of sustainable alternative to fossil-based polymers. In a larger view, integrating renewable feedstocks into the economy could lower crude oil demand, thus limiting economic downturns in the chemical industry due to oil price volatility [4,5]. 

On the other hand, a relevant aspect of using waste as feedstock for PHA production is the possibility to find persistent and ubiquitous pollutants such as polycyclic aromatic hydrocarbons (PAHs) that can accumulate into the final product. PAHs comprise several hundreds of chemically related compounds with various structures [6], constituted by two or more polycondensed aromatic rings containing only carbon and hydrogen atoms. They are mainly formed during the incomplete combustion of various organic materials (e.g., oil, coal, natural gas, biomass and fossil fuels) containing saturated hydrocarbons [7]. Although anthropogenic emissions (e.g., petroleum refinery activities, asphalt, coke and aluminum production, residential heating, coal gasification and liquefying plants) predominate, PAHs can originate from natural sources too (e.g., open burning, natural losses or seepage of petroleum or coal deposits and volcanic activities) [6].

PAHs are highly persistent, hydrophobic and bioaccumulative compounds. Their physical and chemical properties are determined by their conjugated π-electron systems, which are dependent on the number of aromatic rings and their molecular weight [8]. Their water solubility decreases with an increase in the ring number, and although most PAHs are emitted to the atmosphere, their higher affinity for sediments and biota than water make sediments and soils the major environmental sinks for these compounds [9]. 

In addition to being harmful for both aquatic and terrestrial ecosystems, some PAHs are known to possess carcinogenic, mutagenic and teratogenic activities in organisms, including humans, as also assessed by the International Agency for Research on Cancer [10]. They have been associated with various types of cancer; their genotoxic and carcinogenic character is related to the formation of diol epoxides covalently bound to DNA. Furthermore, PAHs can suppress the immune system and are suspected endocrine disrupters [8]. For this reason, the United States Environmental Protection Agency (US EPA) has identified 16 PAHs as priority pollutants [11]. Although this first list has been adopted by several countries, other priority lists have been identified by other authorities, including the EU [12]. 

Tires, electronics, and toys are most affected by the restriction of PAHs in the EU, German and US. Although there are many PAHs, most regulations, analyses and data reporting focus on only a limited number of PAHs, typically between 14 and 20 [6]. 

PAHs can also reach the food chain [13], and several studies have revealed their presence in municipal solid waste (MSW) [14,15,16,17] and soil around the MSW landfill [18]. Various investigations have revealed the presence of PAHs in waste-derived compost and digestate [15,16,17,19]. The data reported in the end-of-waste criteria technical proposals [20] suggest that all types of composts and digestates contain PAH compounds, generally between trace amount levels and a few mg kg^−1^ dry matter. 

The process under investigation is based on the exploitation of organic wastes of different origins for PHA production in combination with wastewater treatment. These biodegradable polymers could have several applications, including, for example, packaging or films for agricultural use. Therefore, it is important to assess the safety of the produced polyesters, which could contain PAHs unintentionally present in the feedstock, i.e., in the organic waste of either municipal or food processing origin. For this reason, 18 PAHs were determined by gas chromatography-mass spectrometry (GC/MS) analysis in several PHA samples obtained at pilot-scale from two types of feedstock and after different extraction procedures. These include eight PAHs (benz(a)anthracene, crysene, benzo(b)fluoranthene, benzo(k)fluoranthene, benzo(j)fluoranthene, benzo(e)pyrene, benzo(a)pyrene, dibenz(ah)anthracene) for which the Regulation EC/1907/2006 (REACH) [21] has set restrictions on their presence in various articles intended for the general public if any of their rubber or plastic components come into direct as well as prolonged or short-term repetitive contact with the human skin or the oral cavity. Therefore, for articles such as bicycles, golf clubs, racquets, household utensils, trolleys, walking frames, tools for domestic use, clothing, footwear, gloves and sportswear, watch-straps, wrist-bands, masks or head-band, any of the eight listed PAHs has to be <1 mg kg^−1^, whereas for toys, including activity toys and childcare articles, any of the listed PAHs has to be <0.5 mg kg^−1^ (see Table 1 for included compounds). Although there is currently no specific PHA regulation, our results were compared with current regulations and guidelines for similar materials and/or applications, and detected PAH values were below threshold limits. To the best of the author’s knowledge, this is the first investigation of the presence of PAHs in several PHA samples from different sources and processing steps.

## 2. Results and Discussion

### 2.1. Method Performance

Because certified reference materials for this combination of analytes and matrix, as well as PHA blank samples, were not available, first, the analytical method performance, which was previously assessed [22], was verified using a perdeuterated standard mix. The recoveries at 50 μg kg^−1^ spiking level were between 60 and 65% for the two lower molecular weight PAHs, namely, naphthalene and acenaphthylene, and between 95 and 100% for the other PAHs. Method limits of detection (MLODs) were 1 μg kg^−1^ for most analytes, and 2 μg kg^−1^ for acenaphthene, fluorene, phenantrene, anthracene, chrysene and benzo[ghi]perylene. The method limits of quantitation (MLOQs) were 5 μg kg^−1^ for all the analytes but for acenaphthene, fluorene, phenantrene and anthracene, the MLOQs were 10 μg kg^−1^.

Before and after each sample batch, a standard solution containing all the PAHs and an instrumental blank were run. 

### 2.2. PAH Contamination in PHA Samples

Results of PAHs determination in the whole set of PHA samples are reported in the following table and graphs as an average; relative standard deviations were below 11% for all the sample typologies. As reported in Table 1, the PAH concentration is variable depending on the type of PAH and the way the PHA sample has been obtained.

Although there is no data availability in the literature regarding PAH determination in biobased plastics specially derived from waste, it is possible to compare the present results with a very recent study conducted on traditional plastics. Kida et al. [23] determined the presence of PAHs in plastics (including polyvinyl chloride, PVC) and rubbers debris of different items, for example, PVC gaskets and rubber tyres. After acetone extraction, the concentration of acenaphtylene, phenanthrene, fluoranthene and pyrene in PVC was 14.30, 2.64, 10.68 and 55.54 mg kg^−1^, respectively; the concentration of anthracene, phenanthrene, fluoranthene and pyrene in rubber was 11.64, 5.63, 13.30 and 28.38 mg kg^−1^, respectively. In comparison with PAH concentration determined in thermal-stabilized raw biomass, which showed the highest PAH content among the whole sample set (Table 1), the PAH content found in PVC and rubber [23] was almost 3 orders of magnitude higher, with the only exception of phenantrene concentration, which was higher in PHA samples (8.3 mg kg^−1^).

**Figure 1:**
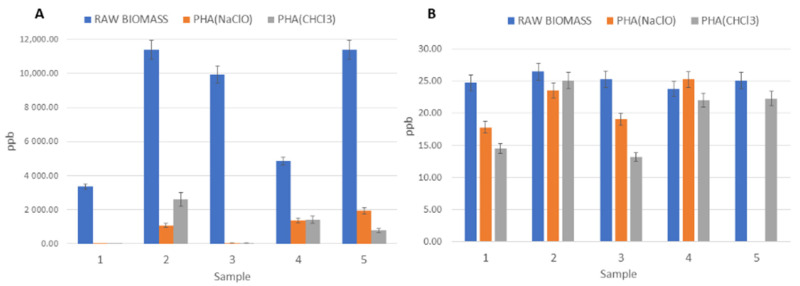
(**A**) Fluoranthene concentration in thermal-stabilized PHA-rich biomass from Treviso facility determined on 5 samples of raw biomass and after chloroform and hypochlorite extraction; (**B**) benz(a)anthracene concentration in thermal-stabilized PHA-rich biomass from Treviso facility determined on 5 samples of raw biomass and after chloroform and hypochlorite extraction.

**Figure 2:**
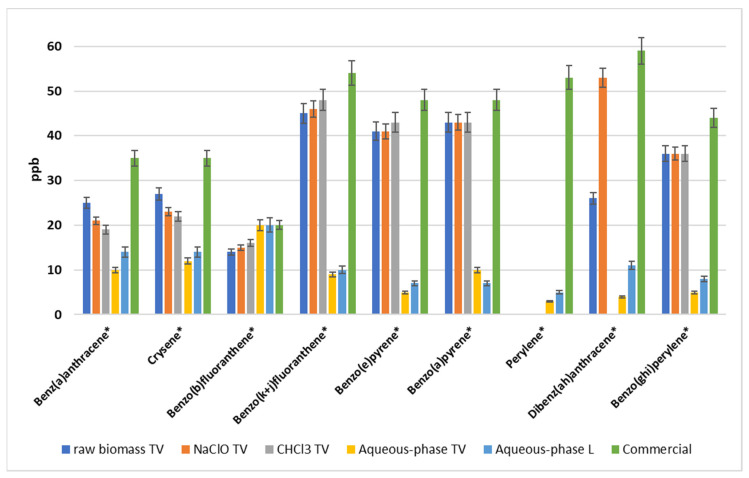
Concentration of PAHs in PHA samples of different origins and after different extraction methods. Limits for these starred PAHs are reported in the European Regulation EC/1907/2006 [21].

Focusing on thermal-stabilized dry PHA-rich biomass, the naphthalene was present at the highest concentration (around 50 mg kg^−1^) in the raw biomass (no extraction applied) and then, because of the extraction with either chloroform or hypochlorite, naphthalene concentration decreased by almost 3 orders of magnitude. This behavior was also evident for other PAHs, more in detail for acenaphthylene, fluorene, phenanthrene, pyrene and fluoranthene. The latter particularly showed a strong decrease in concentration after chloroform and hypochlorite extraction, and the same trend occurred for the five samples of raw PHA-rich biomass that underwent both extraction procedures. As an example, the results of fluoranthene level variation after extraction for thermal-stabilized samples are reported in Figure 1A. On the contrary, in Figure 1B, a different trend of benz(a)anthracene concentration is reported for the same five samples. Indeed, in the ppb range (µg kg^−1^), no significant decrease after extraction occurred for contaminants such as benz(a)anthracene, crysene, benzo(b)fluoranthene, benzo(k + j)fluoranthene, benzo(e)pyrene, benzo(a)pyrene, perylene, dibenz(ah)anthracene, indeno(123cd)pyrene and benzo(ghi)perylene. All these PAHs are below 10 ppm (mg kg^−1^) and most are in the ppb range (µg kg^−1^). Most importantly, the eight most critical PAHs (starred in Table 1 and Figure 2), for which the Regulation EC/1907/2006 (REACH) [21] has set restrictions, are well below the threshold value, i.e., at least 2 orders of magnitude less. Considering again the eight PAHs under restriction, a comparison can be made among the stabilization methods: as shown in Figure 2, the aqueous-phase extraction conducted only on acid-stabilized wet biomass (both from Treviso and Lisbon, yellow and light-blue columns, respectively) allows us to decrease the contaminants level more than chloroform and hypochlorite extractions conducted on thermal-stabilized biomass (grey and orange columns, respectively); therefore, the acid stabilization helped to partially remove contaminants. Moreover, considering the different feedstocks used for PHA production in Treviso and Lisbon and the comparable results obtained after aqueous-phase extraction, it can be concluded that acid stabilization followed by the optimized extraction method leads to the lowest concentration, regardless of the type of feedstock used. In previous studies conducted on the same PHA samples for determination of heavy metals [24] and polychlorinated biphenyls (PCBs) [25], thermal-stabilized biomass resulted in higher metals and PCB content than the acid-stabilized biomass, confirming the effect of acid stabilization in decreasing contaminant levels. On the other hand, in both studies [24,25], the feedstock type affected the contaminant levels, with the PHA from fruit waste having a lower metal and PCB content than PHA obtained from OFMSW-WAS. 

On the other hand, there is no clear distinction between PAH levels in waste-based PHAs, in general, either from OFMSW-WAS or food processing, and PAH levels in commercial PHAs from crops. Indeed, regarding the eight most relevant PAHs, Figure 2 reports a concentration in commercial PHAs (green column) comparable to that determined in PHA derived from OFMWS–WAS and subjected to thermal stabilization, before and after extraction (blue, orange and grey columns). Furthermore, waste-based PHA samples which underwent acid pretreatment for stabilization and then aqueous-phase extraction had lower PCB levels than commercial PHA samples, and especially if coming from fruit waste [25], while metal content was higher in waste-derived PHA, regardless of the feedstock used [24]. In a recent study, attention was focused on the evaluation of in vitro toxicity of several marketed bioplastics, including PHA, and comparable results to fossil-based plastics were found. An interesting result was the lower chemical content detected in raw material than in the final product, in accordance with the addition of additives during the following downstream processing (e.g., compounding) [26]. Although PHAs were not identified among the analyzed toxic chemicals, the latter is a relevant aspect to be considered in the overall production process and economic evaluation. However, in the present study and in previous studies [24,25], contaminants under restriction determined in PHA samples were well below the threshold limit values; indeed, the total content of the analyzed contaminants for all tested PHA types complied with the current regulations and guidelines, such as the limits in plastic materials based on REACH regulation, including toys. 

The determination of the original PAH content in PHAs is also important because plastics can absorb hydrophobic compounds from the surrounding environment and act as vectors for harmful substances. A very recent study [27] showed some differences in the PAH sorption between conventional and bio-based and biodegradable polymers in the marine environment. The biodegradable polymers (not including PHAs) were less prone to PAH sorption; nonetheless, further studies are needed to elucidate potential contributions of PHA. 

Finally, another recent study exploited the ability of defined mixed cultures of bacteria to use some PAHs as substrate for PHA production, thus proposing an attractive way to convert harmful persistent pollutants into environmentally friendly polymers [28,29].

## 3. Materials and Methods 

### 3.1. PHA Samples

**Figure 3:**
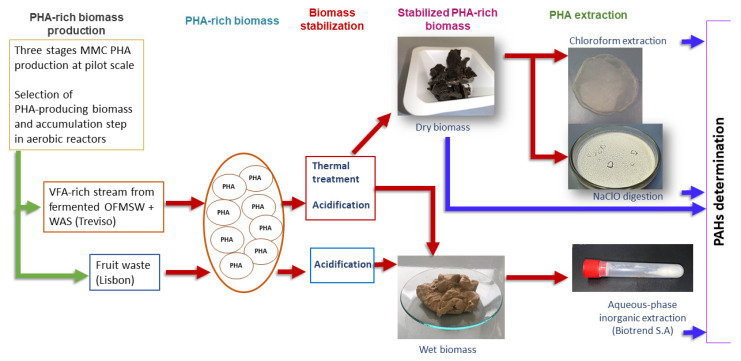
Schematic description of the analyzed samples.

PHA samples were produced at pilot-scale in the REsources from URban BIo-waSte (RES URBIS) project framework, which is aimed to convert several types of urban bio-waste into valuable bio-based products. In this context, two types of feedstock were considered: A) a mixture of the organic fraction of municipal solid waste (OFMSW) and waste activated sludge (WAS) from urban wastewater treatment plant in Treviso municipality (Italy); B) fruit waste collected in Lisbon (Portugal). The description of PHA from Treviso production has been widely reported in [2], while PHA from Lisbon was produced similarly but exploiting a different organic feedstock, i.e., fruit waste. PHA-rich biomass, after the accumulation step, was stabilized for downstream processing following two methods: overnight thermal drying at 60 °C (biomass from Treviso only); acidification with H_2_SO_4_ (biomass from both Treviso and Lisbon). For the type-A) feedstock samples, four different PHA samples were analyzed: (1) raw PHA-rich biomass (dried biomass, no extraction; 5 samples); (2) PHA after extraction from dried biomass with CHCl_3_ (reference method; 5 samples); (3) PHA after oxidation and recovery from dried biomass with NaClO (5 samples); and (4) PHA after extraction from wet biomass (acid storage) with aqueous-phase extraction (4 samples). For the type-B) feedstock samples, one type of PHA sample was analyzed, i.e., PHA after extraction from wet biomass (acid storage) with aqueous-phase extraction reagents (2 samples). Extraction procedures regarding points 2 and 3 (involving CHCl_3_ and NaClO, respectively) are described in a previous work [30], while extraction at points 3 and 4 was conducted by Biotrend S.A. following a reserved protocol. Figure 3 shows a schematic description of the samples and relative treatments.

For comparison, 3 types of commercial PHA, from different producers, that were obtained from fruit waste or crops were analyzed, and their results were expressed as the average. Overall, 24 different PHA samples produced under six different conditions were analyzed. 

### 3.2. PAH Determination

The sample preparation procedure, as well as GC/MS instrumental conditions, were the same as those reported in a previous work [22]. Briefly, PHAs were roughly cut into 2 mm-pieces, and 100 mg-sample was finely dispersed with sand in a 1:8 ratio with the aid of liquid nitrogen. The resulting powder was transferred into a beaker and added with 3 mL of acetonitrile and the deuterated internal standard (d-IS) mix solution. After solvent evaporation (ca. 2 h), the dispersed sample was extracted in 10 mL of hexane with the aid of an ultrasonic bath for 40 min. Surnatant was collected after 20 min centrifugation at 2000× *g*; the procedure was repeated once. The surnatants were loaded into a florisil SPE cartridge, and target compounds were eluted by 6 mL dichloromethane/hexane 1:1 (*v/v*). Solvent was removed by evaporation in a rotary evaporator and then placed under a gentle nitrogen stream up to ca. 20 µL; the residue was redissolved in hexane (final volume 100 µL).

The d-IS mix solution was consisted of fourteen perdeuterated PAHs (chemical purities > 98%, isotopic purities ≥ 98%), concentration 100 µg mL^−1^ in isooctane/toluene 75:25 (*v*/*v*), obtained from Wellington laboratories (Guelph, ON, Canada). This included naphthalene-d8, 2-methylnaphthalene-d10, acenaphthylene-d8, phenanthrene-d10, fluoranthene-d10, benz[a]anthracene-d12, chrysene-d12, benzo[b]fluoranthene-d12, benzo[k]fluoranthene-d12, benzo[a]pyrene-d12, perylene-d12, indeno [1,2,3-c,d]pyrene-d12, dibenz[a,h]anthracene-d14 and benzo[g,h,i]perylene-d12.

PAH determination was carried out as previously reported [22] by an ISQ^TM^ Series Single Quadrupole GC/MS (Thermo Fisher Scientific, St. Peters, MO, USA) equipped with a SLB^TM^-5ms (Supelco, Milan, Italy) fused silica capillary GC column, poly(5% diphenyl/95% dimethyl siloxane) phase, 30 m × 0.25 mm i.d., 0.25 µm film thickness. The carrier gas was He (99.9995% purity). The injector was a programmed-temperature vaporizer (PTV); the injection volume was 1 µL. Temperature programming was set as follows: the initial oven temperature was 40 °C, kept constant for 5 min, then increased to 290 °C at 12 °C min^−1^ and kept constant for 6 min; finally, the temperature was increased to 325 °C with 20 °C min^−1^ and kept constant for 15 min. MS was operated in electron ionization (EI, 70 eV), and acquisition was performed by monitoring the molecular ion of each PAH at its retention time; quantification was carried out using the corresponding perdeuterated isotopologue or a structural omologue perdeuterated isotopologue as IS.

### 3.3. Statistical Analysis

To compare the PAH concentrations in the different samples, analysis of variance (ANOVA) and a Student’s t test were employed in order to identify statistically significant differences. Excel was used to implement the ANOVA and t test functions, and the significance level was set at *p* value 0.05.

## 4. Conclusions

In the context of the circular economy and biobased biodegradable plastics, the present paper provides a PAH profile in PHA samples obtained from organic wastes and biological sludge, still missing in the available literature. In this regard, several PHA samples from different origins and with different process steps were analyzed for their PAH content. Overall, 24 different samples from six different conditions were analyzed. The content of contaminants is generally low, i.e., in the range between ppb and a few ppm. Although there are no available data on raw PHA-rich biomass from fruit waste, it can be concluded that the type of feedstock does not affect the contaminant contents; indeed, both PHAs extracted with aqueous-phase reagents generally show comparable contents. In the commercial PHA, which is derived from crops, PAH concentration is similar to levels found in PHAs derived from OFMSW-WAS.

On the other hand, the type of PHA stabilization and extraction affects the contaminant contents, where acid stabilization and extraction with aqueous-phase reagents cause generally lower contents of the eight regulated PAHs than thermal stabilization and extraction with either hypochlorite or chloroform.

Although a specific regulation does not exist yet, a comparison was made with the regulation and guidelines for similar materials and/or applications. The result was that all tested PHA types, including those analyzed in previous studies [24,25], meet present regulatory standards and guidelines (e.g., limits for Cd and PAHs in plastic materials based on REACH regulation [21]; limits for PCB in Recycling Plastics from Shredder Residue, based on EPA guidelines [28]). Therefore, the use of organic waste as feedstock for PHA production processes appears to be safe for the environment and human health. This is a fundamental aspect for the evaluation of the economic viability of the process, with the view of a possible market use of PHA synthesized from waste.

## Figures and Tables

**Table 1:** Concentration of polycyclic aromatic hydrocarbons (PAHs) in polyhydroxyalkanoate (PHA) samples of different origins. Limits for starred compounds are reported in the European Regulation EC/1907/2006 [21].

PHA Origin	OFMSW–WAS (Treviso)				Fruit Waste (Lisbon)	Commercial
Stabilization	Thermal			Acid	Acid	
Extraction	Raw Biomass	NaClO	CHCl_3_	Aqueous-Phase	Aqueous-Phase	
	(µg kg^−1^) (*n* = 5)	(µg kg^−1^) (*n* = 5)	(µg kg^−1^) (*n* = 5)	(µg kg^−1^) (*n* = 4)	(µg kg^−1^) (*n* = 2)	(µg kg^−1^) (*n* = 3)
Naphthalene	43625	95	66	38372	42400	16728
Acenaphthylene	7210	127	255	209	132	<MLOD
Acenaphthene	8277	3830	3373	170	406	3711
Fluorene	1764	147	220	736	771	176
Phenanthrene	8173	880	963	2555	2660	1121
Anthracene	506	161	123	231	191	124
Fluoranthene	539	58	83	216	210	171
Pyrene	364	49	64	171	137	201
Benz(a)anthracene *	25	21	19	10	14	35
Crysene *	27	23	22	12	14	35
Benzo(b)fluoranthene *	14	15	16	20	20	20
Benzo(k + j)fluoranthene *	45	46	48	9	10	54
Benzo(e)pyrene *	41	41	43	5	7	48
Benzo(a)pyrene *	43	43	43	10	7	48
Perylene *	<MLOD	<MLOD	<MLOD	<MLOQ	5	53
Indeno(123cd)pyrene	38	38	38	<MLOQ	6	43
Dibenz(ah)anthracene *	26	53	<MLOD	<MLOQ	11	59
Benzo(ghi)perylene *	36	36	36	5	8	44

## Data Availability

The data presented in this study are available in article.

## Sample Availability

Samples of polyhydroxyalkanoates are available from the authors.
